# A Distinct Circulating Biomarker Signature of Acute Thrombophlebitis: Associations Among Heparin, Midkine, Pleiotrophin, Dermcidin, and Ghrelin

**DOI:** 10.3390/jcm15197380

**Published:** 2026-09-23

**Authors:** Suna Aydin, İsmail Polat, Kader Ugur, Sefa Şenol, Davut Azboy, Kevser Tural, Suleyman Aydin, Do-Youn Lee

**Affiliations:** 1 Department of Cardiovascular Surgery, Fethi Sekin City Hospital, Elazig 23300, Türkiye; ssenol2012@gmail.com (S.Ş.); drazboy@gmail.com (D.A.); 2 Department of Histology and Embryology, Faculty of Veterinary Medicine, Firat University, Elazig 23119, Türkiye; 3 Department of Cardiology, Fethi Sekin City Hospital, Elazig 23300, Türkiye; drismailpolat49@gmail.com; 4 Department of Internal Medicine (Endocrin, and Metabolic Diseases), Faculty of Medicine, Firat University, Elazig 23119, Türkiye; kaderaksoy06@hotmail.com; 5 Department of Cardiovascular Surgery, Mehmet Akif Ersoy Thoracic and Cardiovascular Surgery Training and Research Hospital, Istanbul 34303, Türkiye; ktrl2011@hotmail.com; 6 Department of Medical Biochemistry, Faculty of Medicine, Firat University, Elazig 23119, Türkiye; saydin1@hotmail.com; 7 College of General Education, Kookmin University, Seoul 02707, Republic of Korea

**Keywords:** thrombophlebitis, dermcidin, ghrelin, midkine, pleiotrophin, heparin, biomarkers, inflammation

## Abstract

**Background:** Acute superficial venous thrombosis (SVT) presenting with clinical features of thrombophlebitis is increasingly recognized as a thromboinflammatory disorder in which innate immunity and coagulation are closely interconnected. Nonetheless, the role of endogenous antimicrobial peptides and heparin-binding growth factors in its pathogenesis is still inadequately comprehended. We investigated circulating dermcidin (DCD), ghrelin (GHR), heparin (HP), midkine (MK), and pleiotrophin (PTN) to explore their potential involvement in the thromboinflammatory network underlying SVT. **Methods:** In this prospective case–control study, 30 individuals with SVT and 28 healthy controls matched for age and sex were recruited. DCD, GHR, HP, MK, and PTN concentrations were quantified using enzyme-linked immunosorbent assays. **Results:** Patients with SVT exhibited significantly higher circulating DCD, GHR, MK, and PTN concentrations and significantly lower the measured HP concentration than healthy controls (all *p* < 0.05). Following LMWH treatment, the measured HP concentration increased significantly (*p* < 0.05), whereas DCD, GHR, MK, and PTN showed downward trends. **Conclusions:** Acute superficial venous thrombosis is associated with coordinated alterations in circulating the measured heparin, dermcidin, ghrelin, midkine, and pleiotrophin, reflecting a systemic thromboinflammatory biomarker profile. These findings suggest potential interactions among heparin biology, heparin-binding growth factors, and innate immune mediators but do not establish direct molecular interactions, heparin sequestration, functional depletion, or causality. This biomarker profile establishes a foundation for creating hypotheses for forthcoming investigations that include direct biochemical validation, standardized longitudinal sampling, and independent cohorts.

## 1. Introduction

Acute thrombophlebitis is a common thromboinflammatory vascular disorder characterized by the coexistence of venous thrombosis and inflammation of the vessel wall. It predominantly affects the superficial veins of the lower extremities and represents a significant clinical burden because of pain, edema, impaired mobility, and the risk of progression to deep vein thrombosis or pulmonary embolism. The incidence of thrombophlebitis varies according to age, sex, and the presence of acquired or inherited thrombotic risk factors [1]. Women are more frequently affected during their reproductive years owing to pregnancy, hormone replacement therapy, and oral contraceptive use, whereas the incidence rises substantially in both sexes with advancing age [2]. Population-based studies demonstrate substantial ethnic variation in the incidence of venous thromboembolism, with consistently lower rates reported in East Asian populations than in populations of European or African ancestry, reflecting a complex interplay of genetic susceptibility and environmental risk factors [3,4,5].

Although thrombophlebitis has traditionally been explained by Virchow’s triad—venous stasis, endothelial injury, and hypercoagulability—current evidence suggests that this paradigm alone does not fully explain disease development. Instead, complex interactions among inflammation, endothelial activation, innate immunity, and thrombus formation contribute to disease initiation and progression [1,6]. Increasing evidence indicates that thrombosis arises through complex interactions among endothelial cells, platelets, leukocytes, inflammatory mediators, and coagulation pathways. This integrated process, known as thromboinflammation or immunothrombosis, links innate immune activation with hemostatic dysregulation during vascular injury [7].

Endothelial activation initiates platelet adhesion, leukocyte recruitment, cytokine release, and tissue factor expression, while neutrophil extracellular traps (NETs) and activated monocytes further amplify thrombus formation [1,8]. Beyond these classical mechanisms, endogenous antimicrobial peptides and other innate immune mediators have emerged as important regulators of endothelial homeostasis, inflammatory signaling, angiogenesis, oxidative stress, and coagulation, suggesting that they may actively participate in thromboinflammatory disorders [8,9,10,11,12,13]. In addition to its well-established anticoagulant activity, heparin has emerged as a molecule with antimicrobial and immunomodulatory properties. By binding to bacterial surface proteins, microbial toxins, and host defense peptides, heparin can interfere with pathogen adhesion, biofilm formation, and inflammatory responses, thereby contributing to innate host defense [14]. However, the molecular networks integrating innate immunity with thromboinflammation in acute thrombophlebitis remain poorly understood. Elucidating these pathways may provide novel mechanistic insights and facilitate the development of clinically relevant biomarkers and therapeutic strategies.

Among bioactive molecules implicated in vascular inflammation, dermcidin (DCD), ghrelin (GHR), heparin (HP), midkine (MK), and pleiotrophin (PTN) have attracted increasing attention because of their pleiotropic roles in regulating inflammation, endothelial function, angiogenesis, oxidative stress, innate immunity, and coagulation. Despite their distinct molecular origins, these mediators converge on key biological processes, including innate immunity, inflammatory regulation, endothelial homeostasis, angiogenesis, tissue remodeling, and coagulation, suggesting that they constitute components of an integrated thromboinflammatory network [10,15,16,17].

DCD is a constitutively expressed antimicrobial peptide that forms part of the innate host defense system [10]. Beyond its antimicrobial activity, DCD has been linked to endothelial dysfunction, oxidative stress, platelet activation, and thrombin generation, suggesting a role in thrombotic disorders [18]. In contrast, GHR, originally identified as a metabolic hormone, is now recognized as a multifunctional peptide with anti-inflammatory, antioxidant, endothelial-protective, and antimicrobial properties that may limit excessive vascular inflammation and preserve endothelial integrity [19].

Besides its well-established anticoagulant activity, heparin regulates inflammatory signaling through interactions with cytokines, chemokines, complement components, selectins, adhesion molecules, and numerous heparin-binding proteins, thereby modulating leukocyte recruitment and endothelial activation [20]. Among these, MK and PTN are structurally related heparin-binding growth factors that are rapidly induced following tissue injury and participate in inflammation, angiogenesis, tissue repair, and vascular remodeling [21].

The strong attraction of MK and PTN to heparin and heparan sulfate indicates mutual interactions that could affect the biological accessibility and function of both ligands in the vascular microenvironment [21]. Supporting this concept, our recent study in patients with deep vein thrombosis demonstrated significantly decreased circulating the measured heparin concentration together with elevated MK and PTN levels, suggesting disruption of heparin homeostasis during thromboinflammation [22]. Whether a similar molecular network exists in acute thrombophlebitis, however, remains unknown.

To the best of our knowledge, this is the first study to simultaneously evaluate circulating DCD, GHR, HP, MK, and PTN in patients with acute thrombophlebitis. Although each molecule has individually been associated with innate immunity, vascular inflammation, or thrombosis, their coordinated behavior within a unified thromboinflammatory framework remains poorly understood [23]. Emerging evidence suggests that HP functions as a central regulator of vascular homeostasis through its interactions with MK and PTN [22]. Increased concentrations of these growth factors may reduce the bioavailability of free HP, thereby promoting a prothrombotic and proinflammatory vascular milieu, while concurrent alterations in DCD and GHR may reflect the activation of complementary host-defense pathways. Together, these observations support the concept of a HP-binding thromboinflammatory network, linking antimicrobial peptides, HP, and heparin-binding growth factors to vascular inflammation and thrombosis [8,9,10,11,12,13,14].

We posited that acute thrombophlebitis is defined by synchronized changes in circulating DCD, GHR, HP, MK, and PTN. Accordingly, this prospective study simultaneously quantified these biomarkers in patients with acute superficial venous thrombosis (SVT) presenting with clinical features of thrombophlebitis and healthy controls, examined their interrelationships, and evaluated the potential contribution of the MK–PTN–HP axis to disease pathophysiology. By integrating antimicrobial peptides with HP biology and heparin-binding growth factors, this study proposes a novel mechanistic framework linking innate immunity and coagulation in SVT and provides a foundation for future biomarker development and targeted therapeutic strategies.

## 2. Materials and Methods

### 2.1. Study Population: Inclusion and Exclusion Criteria

This prospective case–control study was conducted at the Department of Cardiovascular Surgery, Elazığ Fethi Sekin City Hospital, Elazığ, Türkiye. A total of 30 consecutive patients with objectively confirmed acute superficial venous thrombosis (SVT) who presented to the outpatient clinic were enrolled during the study period (9 July 2026 to 20 August 2026). Recruitment and biological sample collection commenced only after formal approval had been obtained from the relevant Ethics Committee and the official approval letter had been issued. The study protocol was reviewed and approved by the Fırat University Non-Interventional Research Ethics Committee (Decision No.: 2026/10-61 and date of approval: 9 July 2026). All procedures involving human participants were conducted in accordance with the ethical principles of the Declaration of Helsinki and its subsequent amendments (the latest version (2024 revision)).

Written informed consent was obtained from all participants before enrollment in the study. The control group comprised 28 healthy volunteers matched for age, sex, and body mass index (BMI), with no history of systemic disease, acute infection, inflammatory disease, thrombotic disorders, or regular medication use.

Patients were diagnosed with SVT when they exhibited characteristic clinical features, including localized pain, swelling, erythema, increased skin temperature, tenderness along the affected vein, and occasionally ecchymosis. In cases of superficial thrombophlebitis, the affected vein typically appeared as a tender, palpable cord with linear induration corresponding to the course of the superficial venous system [1]. All 30 patients included in the thrombophlebitis group had objectively confirmed SVT. Doppler ultrasonography was performed in all patients, and SVT was confirmed by the presence of intraluminal thrombotic material and non-compressibility of the affected superficial vein. The study specifically included patients with acute SVT presenting with clinical features of thrombophlebitis and did not include patients with deep venous thrombosis or other forms of venous thromboembolism [1]. None of the patients required surgical intervention. Enoxaparin, a low-molecular-weight heparin (LMWH), was administered subcutaneously to all patients at a dose of 40 mg once daily as part of the routine conservative treatment protocol for SVT, which is typically continued for three months.

Inclusion Criteria: Patients who had not previously received treatment for the present episode prior to admission, were at least eighteen years old, and had a clinical diagnosis of SVT confirmed by Doppler ultrasonography (Doppler USG) were included. All participants were required to provide informed consent and be available for blood sampling before and after routine LMWH therapy. Healthy controls were included if they were age, sex, and BMI-matched, had no history of thromboembolic or systemic disease, and had normal venous Doppler findings.

Exclusion Criteria: Participants were excluded if they had a history of chronic venous insufficiency, recurrent thrombophlebitis, previous venous thromboembolism, or any documented arterial thrombotic event. Medical histories, comorbidities, medication use, and previous thromboembolic diagnoses were verified through the National Health Information System and hospital electronic medical records, in addition to patient interviews.

Individuals receiving anticoagulant, antiplatelet, corticosteroid, immunosuppressive, or hormone replacement therapy within the preceding three months were excluded, as were patients with active malignancy or a history of cancer treatment within the previous five years. Patients with known hereditary or acquired thrombophilia, including protein C deficiency, protein S deficiency, antithrombin deficiency, Factor V Leiden mutation, prothrombin G20210A mutation, or antiphospholipid syndrome, were also excluded.

Additional exclusion criteria comprised acute or chronic systemic inflammatory, autoimmune, or infectious diseases, including rheumatoid arthritis, systemic lupus erythematosus, inflammatory bowel disease, sepsis, or any febrile illness unrelated to thrombophlebitis at the time of blood sampling. Individuals with severe hepatic or renal dysfunction, pregnancy or lactation, recent major surgery or significant trauma within the previous three months, inadequate clinical records, unsuitable or hemolyzed blood samples, or refusal or inability to provide written informed consent were likewise excluded.

### 2.2. Blood Sample Collection and Processing

In the present study, peripheral venous blood samples (5 mL) were obtained from all volunteer participants under fasting conditions as part of routine clinical follow-up, following the previously described venous blood sampling and good laboratory practice procedures [24,25]. In patients with SVT, samples were collected immediately before initiation of LMWH therapy and subsequently between days 5 and 10 of treatment, whereas a single sample was obtained from each healthy control under the same fasting conditions. Blood was collected into tubes containing aprotinin as a protease inhibitor to minimize peptide degradation and preserve biomarker stability. Samples were centrifuged at 4.000 rpm for 5 min to separate plasma, which was subsequently aliquoted into sterile polypropylene microcentrifuge (Eppendorf, Singapore) tubes and stored at −20 °C until biochemical analyses. Identical preanalytical procedures were applied to samples from patients and controls [24,25].

### 2.3. Biochemical Analyses

Plasma concentrations of DCD, GHR, HP, MK, and PTN were quantified using commercially available enzyme-linked immunosorbent assay (ELISA) kits according to the manufacturers’ instructions and previously published analytical protocols [24,25]. HP, MK, and PTN concentrations were measured using human-specific ELISA kits (Shanghai Sunredbio Technology Co., Ltd., Shanghai, China) with the following specifications: HP (Cat. No. 201-12-1130; analytical sensitivity: 0.05 ng/mL; assay range: 0.05–15 ng/mL), MK (Cat. No. 201-12-7339; analytical sensitivity: 4.006 ng/L; assay range: 5–1500 ng/L), and PTN (Cat. No. 201-12-0163; analytical sensitivity: 7.118 ng/mL; assay range: 8–2200 ng/mL).

DCD concentrations were determined using a human-specific ELISA kit (Cloud-Clone Corp., Houston, TX, USA; Cat. No. SEC896Hu), with an assay range of 0.8–50 ng/mL and a minimum detectable concentration of 0.29 ng/mL. Plasma GHR concentrations were measured using a commercially available human Acylated Ghrelin ELISA kit (Cat. No. A05106; SPI-Bio/Bertin Pharma, Montigny-le-Bretonneux, France), which had an assay range of 2–250 pg/mL, an analytical sensitivity of 0.3 pg/mL, and a plasma limit of detection of <5 pg/mL, according to the manufacturer’s specifications. Whenever analyte concentrations exceeded the upper limit of the standard curve or when otherwise required, plasma samples were diluted with 0.9% sterile physiological saline, and the measured concentrations were multiplied by the corresponding dilution factor to obtain the final values.

According to the manufacturers’ specifications, the intra-assay coefficient of variation (CV) was <10% and the inter-assay CV was <12% for all ELISA kits, indicating acceptable analytical precision and reproducibility. All measurements were performed in accordance with the manufacturers’ protocols, with laboratory personnel blinded to the clinical status of the participants; all samples were analyzed as single determinations, with no duplicate or repeat analyses performed. Following the addition of the stop solution to each well, optical density (OD) was measured at 450 nm within 10 min using a ChroMate microplate reader (Awareness Technology Inc., Palm City, FL, USA). Plasma concentrations of DCD, GHR, HP, MK, and PTN were calculated from analyte-specific standard curves according to the manufacturers’ instructions. All washing steps were performed using a BioTek ELx50 automatic microplate washer (BioTek Instruments Inc., Winooski, VT, USA).

### 2.4. Statistical Analysis

Statistical analyses were performed using IBM SPSS Statistics for Windows, Version 25.0 (IBM Corp., Armonk, NY, USA). Continuous variables were expressed as mean ± standard deviation (SD), whereas categorical variables were presented as numbers (n) and percentages (%). Data distribution was assessed using the Shapiro–Wilk test. Comparisons between patients with SVT thrombophlebitis and healthy controls were performed using the Mann–Whitney U test, while within-patient comparisons of biomarker concentrations before and after LMWH treatment were performed using the Wilcoxon signed-rank test. Associations among circulating DCD, GHR, HP, MK, and PTN concentrations were assessed using Spearman’s rank correlation coefficient. All statistical tests were two-tailed, and *p* < 0.05 was considered statistically significant.

The preparation and graphical design of Figure 1, as well as language editing, grammar correction, and manuscript polishing, were all aided by ChatGPT (OpenAI, GPT-5.6 Luna). All AI-assisted content (GPT-5.6 Luna) was examined and authorized by the authors, who are solely responsible for the finished product.

## 3. Results

The biological functions of ghrelin, dermcidin, heparin, pleiotrophin, and midkine in acute superficial venous thrombosis are comprehensively summarized in Figure 1. These molecules provide a conceptual framework for understanding the changes in innate immunity, endothelial function, coagulation, and tissue remodeling that were identified in the current investigation. It also shows how these mediators may interact in inflammation, coagulation, endothelial function, and tissue remodeling during acute superficial venous thrombosis.

A total of 30 patients with SVT and 28 healthy controls were included in the study. Baseline demographic and clinical characteristics are summarized in Table 1. There were no significant differences between the patient and control groups with respect to age, BMI, or smoking status (all *p* > 0.05). Among patients, 56.7% were female. The most frequent predisposing factor was the postpartum period (40.0%), followed by idiopathic thrombophlebitis (36.7%), cardio-renal-metabolic comorbidities (16.7%), and trauma (6.6%) (Table 2). Pain accompanied by swelling was the predominant presenting symptom (53.3%), whereas the left lower extremity was the most commonly affected site (63.3%) (Table 3).

Circulating HP concentrations were significantly lower in patients with acute SVT before treatment than in healthy controls (1.47 ± 0.430 vs. 3.63 ± 1.91 ng/mL, *p* < 0.001). Following LMWH therapy, HP concentrations increased significantly to 2.03 ± 0.44 ng/mL compared with pre-treatment levels (*p* < 0.001), but remained significantly lower than those observed in the control group (*p* < 0.001) (Figure 2). In contrast, circulating DCD, GHR, MK, and PTN concentrations were significantly higher in patients than in healthy controls (all *p* < 0.05) (Figure 3, Figure 4, Figure 5 and Figure 6). After LMWH treatment, all four biomarkers exhibited downward trends (all *p* < 0.05). Overall, SVT was characterized by a distinct circulating biomarker profile comprising decreased HP together with increased DCD, GHR, MK, and PTN. LMWH treatment significantly reduced DCD, GHR, MK, and PTN levels. Notably, post-treatment MK levels decreased to values even lower than those observed in the control group. However, GHR and PTN levels declined following treatment to levels that were no longer significantly different from those of the control group.

Correlation analysis identified several significant relationships among the investigated parameters. A complete summary of all correlation analyses, including both significant and non-significant correlations, is provided in Table 4. The presence of HP and DCD (*r* −0.599; *p* 0.001) and HP and GHR levels (*r* −0.563, *p* 0.001) showed a significant moderate–strong negative correlation. There was a moderate–strong positive correlation between presence of DCD–GHR (*r* +0.399; 0.001), and DCD–PTN (*r* +0.042, *p* 0.042), and GHR–MK (*r* +0.418; *p* 0.001), and GHR–PTN (*r* +0.321, *p* 0.002). The remaining parameters do not significantly correlate with one another (Table 4).

To facilitate interpretation of these findings, a Proposed Mechanistic Model was developed by integrating the observed biomarker alterations with current biological evidence (Figure 7). The model depicts a putative heparin-binding thromboinflammatory network in which coordinated dysregulation of DCD, GHR, HP, MK, and PTN may contribute to endothelial dysfunction, inflammatory activation, impaired anticoagulant homeostasis, angiogenic imbalance, and thrombus formation in thrombophlebitis. By illustrating potential molecular crosstalk among these mediators, the framework provides a biological context for the present findings and identifies candidate pathways that may serve as future diagnostic biomarkers or therapeutic targets. Importantly, this schematic is intended solely as a hypothesis-generating conceptual model and should not be interpreted as evidence of direct causal relationships.

## 4. Discussion

Thrombophlebitis is increasingly recognized as a thromboinflammatory vascular condition in which the interrelated processes encompassed by Virchow’s triad—endothelial dysfunction or injury, venous stasis, and hypercoagulability—converge with innate immune and inflammatory pathways to shape thrombus formation and vascular inflammation [26]. Although these mechanisms are well established at the clinical level, the molecular mediators linking coagulation, inflammation, and host defense remain incompletely understood. The current research uncovers a previously overlooked biomolecular signature marked by diminished circulating HP and heightened concentrations of DCD, GHR, MK, and PTN in individuals with SVT, indicating a synchronized thromboinflammatory network that encompasses anticoagulant depletion and innate immune activation.

A key finding of the present study was the marked reduction in circulating heparin (HP) concentrations during acute superficial venous thrombosis (SVT), followed by a significant increase after treatment with low-molecular-weight heparin (LMWH). Beyond its anticoagulant activity, heparin is a multifunctional glycosaminoglycan with recognized anti-inflammatory and antimicrobial properties [14,27,28]. The reduction in circulating HP may therefore reflect altered heparin availability or handling during acute thromboinflammation. However, because we measured circulating HP concentrations without assessing free versus protein-bound fractions, tissue distribution, or direct molecular interactions, the biological basis of this alteration cannot be determined from the present data.

The concomitant elevation in midkine (MK) and pleiotrophin (PTN), both high-affinity heparin-binding growth factors, provides a biologically plausible context for this observation. Their inverse relationship with circulating HP raises the possibility that altered interactions between heparin and heparin-binding proteins may influence heparin homeostasis during vascular inflammation. Nevertheless, this interpretation remains hypothesis-generating. The present study does not demonstrate direct HP–MK/PTN binding, heparin sequestration, or functional depletion of heparin. Moreover, the increase in measured HP following LMWH treatment cannot be interpreted as evidence of restoration of the measured heparin concentration because exogenous anticoagulant administration and the subsequent resolution of the acute inflammatory state may both contribute to the observed change. Moreover, the observed post-treatment increase in measured HP concentration should be interpreted cautiously, as the present study does not allow us to determine whether this change reflects an alteration in endogenous HP levels, an effect related to LMWH treatment, or other factors influencing the measured HP signal. Therefore, the observed increase should not be interpreted as evidence of increased endogenous heparin. Direct biochemical and functional studies will therefore be required to determine whether HP–MK/PTN interactions have biological consequences in SVT.

Our previous study in patients with deep vein thrombosis similarly demonstrated decreased HP together with increased MK and PTN concentrations [22]. The consistency of these observations across venous thrombotic conditions supports the possibility that coordinated alterations in heparin-associated biology may accompany thromboinflammation. Importantly, however, this concordance does not establish a causal pathway. MK and PTN have pleiotropic effects on endothelial responses, leukocyte recruitment, tissue remodeling, and innate immune signaling [13,21]. Their elevation may consequently represent either an active component of vascular inflammation or a response to tissue injury and thrombotic activation.

Dermcidin (DCD) was also elevated during acute SVT. As an antimicrobial peptide with reported links to platelet activation and prothrombotic signaling [18,29], increased DCD may reflect activation of host-defense pathways that intersect with thromboinflammatory processes. Similarly, increased circulating ghrelin may indicate broader metabolic and vascular responses to acute disease. Although ghrelin has established endocrine functions, emerging evidence suggests effects on inflammatory and endothelial pathways [19,30]. The present data cannot distinguish between a compensatory response, a consequence of systemic inflammation, and a nonspecific marker of acute disease. These findings should therefore be interpreted as biomarker associations rather than evidence of protective or pathogenic effects.

An additional, potentially relevant layer is extracellular vesicle (EV)-mediated intercellular communication. EVs can transport proteins, lipids, and nucleic acids and influence endothelial function, inflammation, coagulation, and circulating biomarker profiles. Recent work has emphasized their multifaceted role in vascular and inflammatory biology and their potential value in biomarker development [31]. Although EVs were not assessed in this study, EV-mediated signaling could provide one biological context linking cellular activation with the coordinated circulating changes observed here. This concept is yet hypothetical and has to be investigated using functional endothelium and coagulation assays, molecular interaction investigations, and integrated EV profiling. Collectively, these findings support a circulating thromboinflammatory biomarker profile in acute SVT rather than a definitively established mechanistic network. The coordinated alterations in HP, MK, PTN, DCD, and ghrelin identify interconnected biological pathways worthy of further investigation while highlighting the need for direct mechanistic validation.

An important consideration is the etiological heterogeneity of the study population. The cohort included postpartum, idiopathic, trauma-related, and cardio-renal-metabolic cases, each of which may be associated with distinct inflammatory, metabolic, endothelial, and thrombotic processes. Such heterogeneity is a possible source of residual biological confounding and may have contributed to inter-individual variability in circulating DCD, GHR, HP, MK, and PTN concentrations. Therefore, rather than an etiology-specific signature, the current data should be understood as representing an overall biomarker profile linked to acute SVT.

The observed elevation in circulating ghrelin should likewise be interpreted cautiously. Although ghrelin has established metabolic and endocrine functions and has been implicated in inflammatory and endothelial processes [19,30], the present observational design cannot determine whether its increase represents a compensatory response, a consequence of acute thromboinflammation, or a broader metabolic response to illness. Therefore, rather than indicating a protective or pathogenic role for ghrelin, the study indicates a relationship with the acute SVT condition.

### Study Limitations and Interpretation

Several limitations should be considered when interpreting the present findings. First, the relatively small sample size and the assessment of multiple biomarkers may have limited statistical power and introduced some uncertainty into the observed estimates. Accordingly, the between-group comparisons and correlation analyses are best regarded as exploratory and should be interpreted cautiously. Although the statistical analyses were reviewed with consideration of multiple comparisons, the observed associations would benefit from confirmation in larger and independently studied cohorts.

Second, methodological considerations related to the ELISA measurements should also be taken into account, particularly regarding the potential for heparin-related cross-reactivity or assay interference. Although the assays were performed according to the manufacturers’ protocols, independent analytical validation specifically addressing potential heparin-related interference was not undertaken in the present study. Our previous work showed that the assay can detect LMWH [22]; however, this does not establish analytical discrimination between administered enoxaparin and endogenous heparin. Therefore, post-treatment HP measurements are more appropriately interpreted as changes in measured HP concentrations during LMWH treatment rather than as specific measures of endogenous heparin.

Third, acute superficial thrombophlebitis was clinically assessed in the cardiovascular surgery outpatient setting, and the study was not designed to provide detailed anatomical characterization of thrombus extent or to evaluate progression to other thrombotic conditions. The findings should therefore be considered within the clinical context of acute superficial thrombophlebitis, with caution regarding their applicability to other thrombotic disorders.

Finally, the temporal changes observed after LMWH treatment should be interpreted with appropriate caution. In the absence of an untreated or alternative-treatment comparator and given the clinically determined timing of post-treatment sampling, the observed changes may reflect treatment-related effects, the natural temporal course of acute superficial thrombophlebitis, or a combination of these factors. Thus, the present findings may provide preliminary evidence of biomarker associations and temporal changes, but they do not by themselves establish a causal or specific molecular mechanism. Larger prospective studies incorporating standardized longitudinal sampling, appropriate comparator groups, and analytically validated assays may help clarify the biological significance and potential clinical relevance of these observations.

## 5. Conclusions

The present study identified differences in circulating concentrations of heparin, midkine, pleiotrophin, dermcidin, and ghrelin in patients with acute superficial venous thrombosis (SVT), together with several associations among these biomarkers. Collectively, these findings may be consistent with alterations in inflammatory, vascular, coagulation-related, and innate immune processes accompanying acute SVT. However, the findings should be interpreted cautiously, particularly in view of the limited sample size, exploratory correlation analyses, and analytical considerations related to the measurement of heparin during LMWH treatment. The observed biomarker associations and temporal changes do not by themselves establish direct molecular interactions, heparin sequestration, functional changes in heparin activity, or a causal role of MK or PTN in SVT.

The potential relationship among MK, PTN, and heparin may therefore represent a hypothesis for further investigation rather than an established mechanistic pathway. Larger prospective studies with standardized longitudinal sampling, appropriate comparator groups, analytically validated assays, and complementary molecular and functional approaches may help clarify the biological significance and potential clinical relevance of these observations.

## Figures and Tables

**Figure 1:**
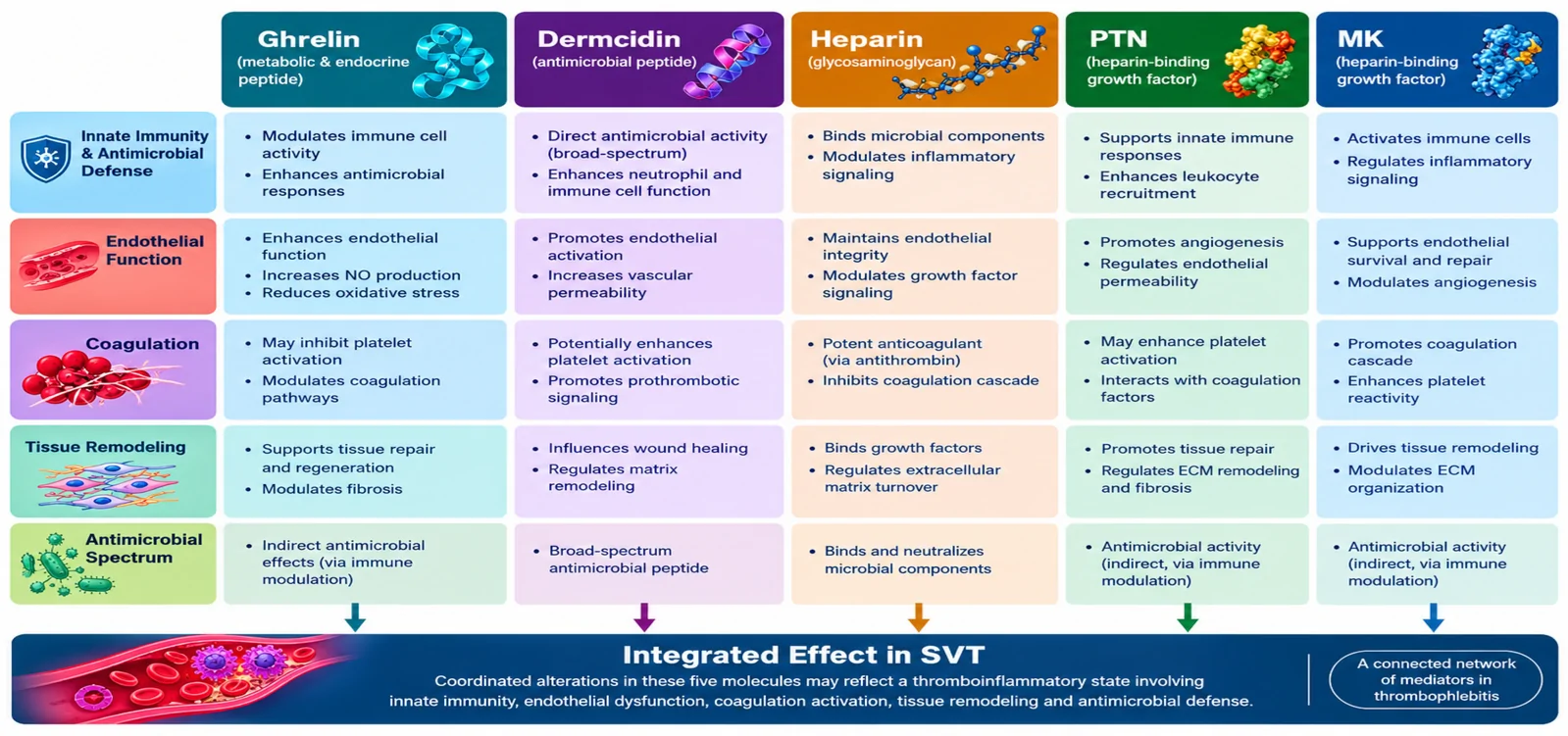
Integrated biological roles of ghrelin, dermcidin, heparin, pleiotrophin, and midkine in acute superficial venous thrombosis (this figure was generated with assistance from ChatGPT (OpenAI) and finalized by the first author).

**Figure 2:**
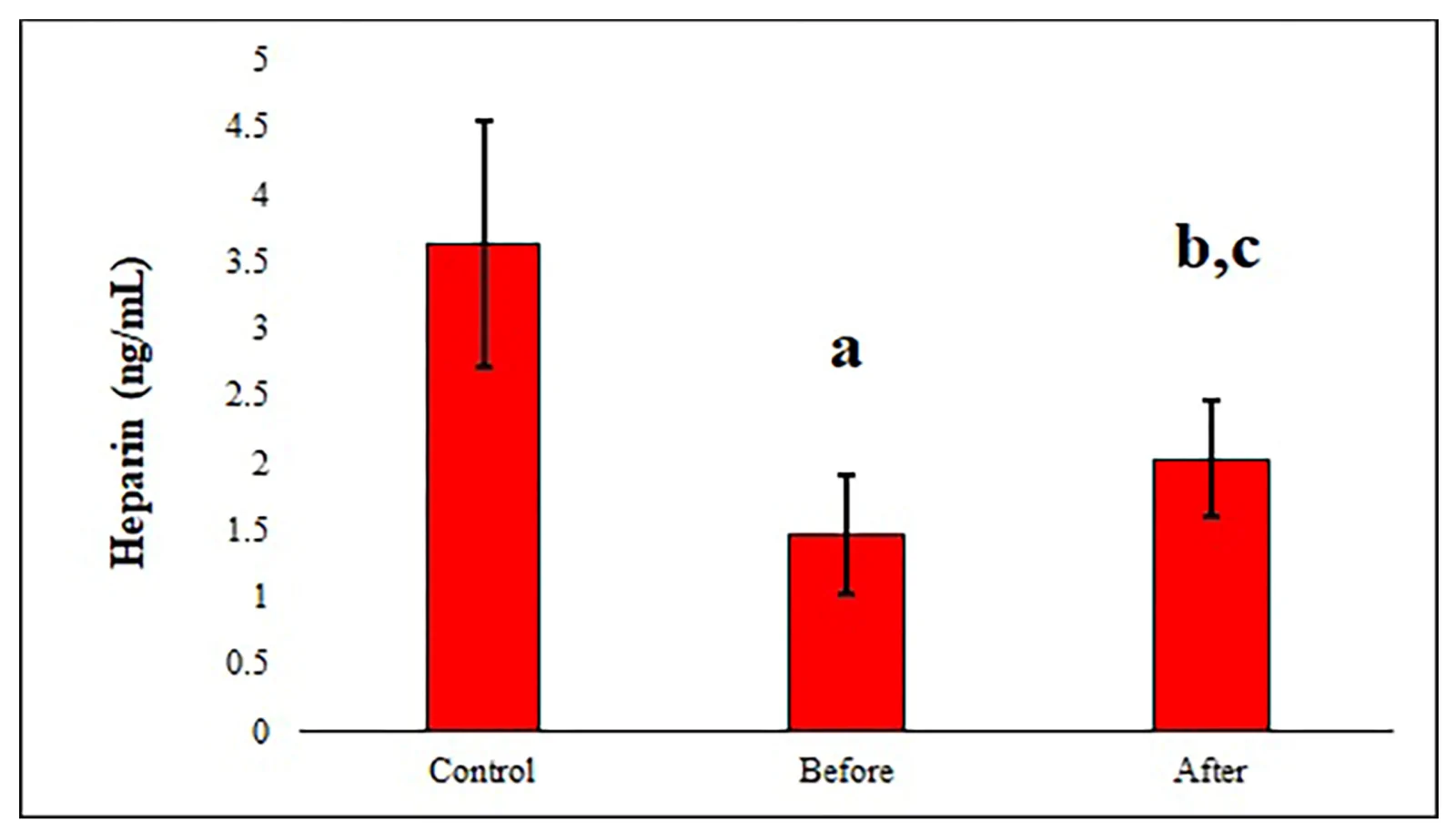
Plasma HP levels in healthy controls and patients with superficial venous thrombosis before and after LMWH. Data are presented as mean ± SD, and error bars represent standard deviations. a: Healthy control group versus pre-treatment group (*p* < 0.001); b: Healthy control group versus post-treatment group (*p* < 0.001); c: Pre-treatment group versus post-treatment group (*p* < 0.001).

**Figure 3:**
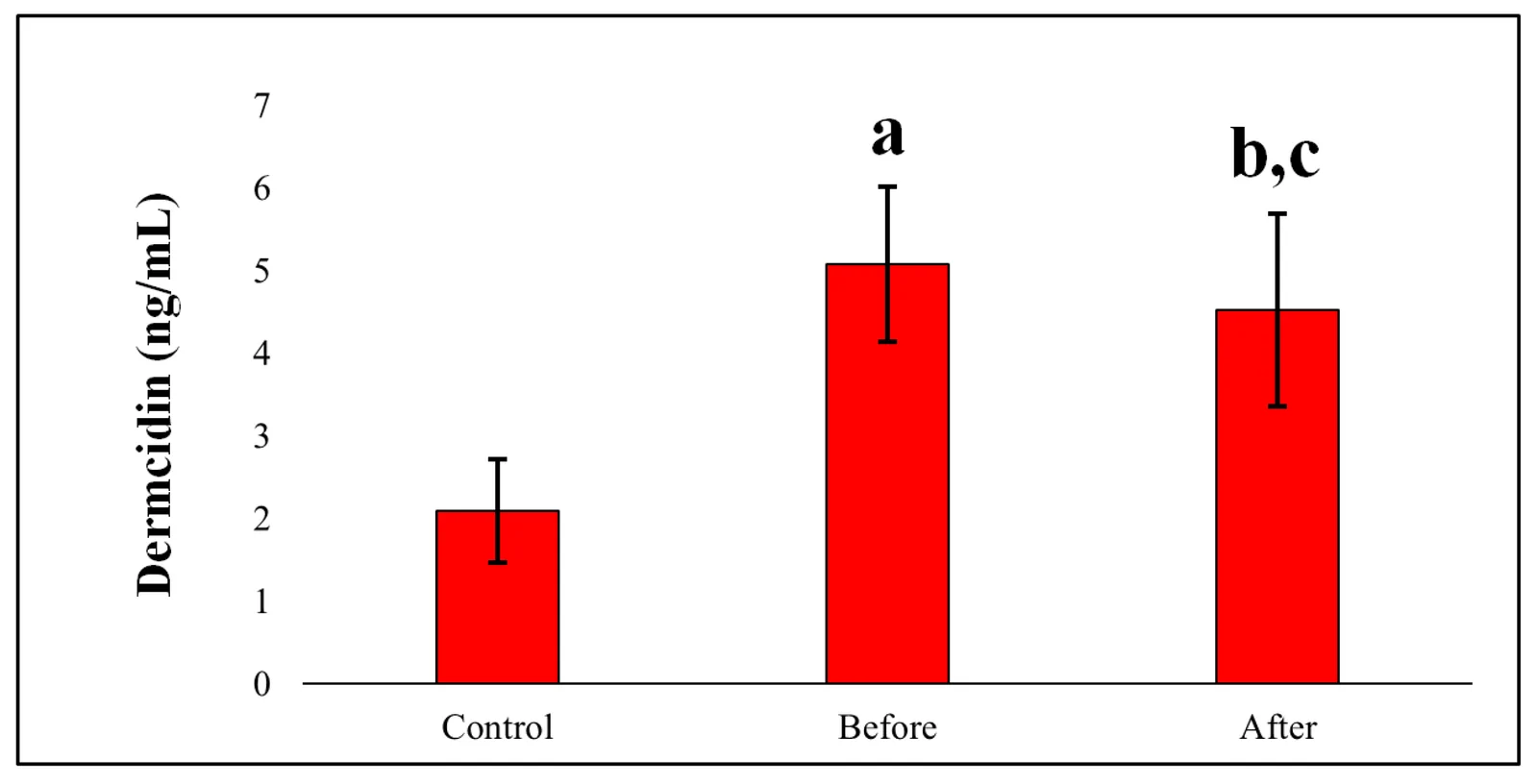
Plasma DCD levels in healthy controls and patients with superficial venous thrombosis before and after LMWH. Data are presented as mean ± SD, and error bars represent standard deviations. DCD: dermcidin. LMWH: low-molecular-weight heparin. a: Healthy control group versus pre-treatment group (*p* < 0.001); b: Healthy control group versus post-treatment group (*p* < 0.001); c: Pre-treatment group versus post-treatment group (*p* = 0.040).

**Figure 4:**
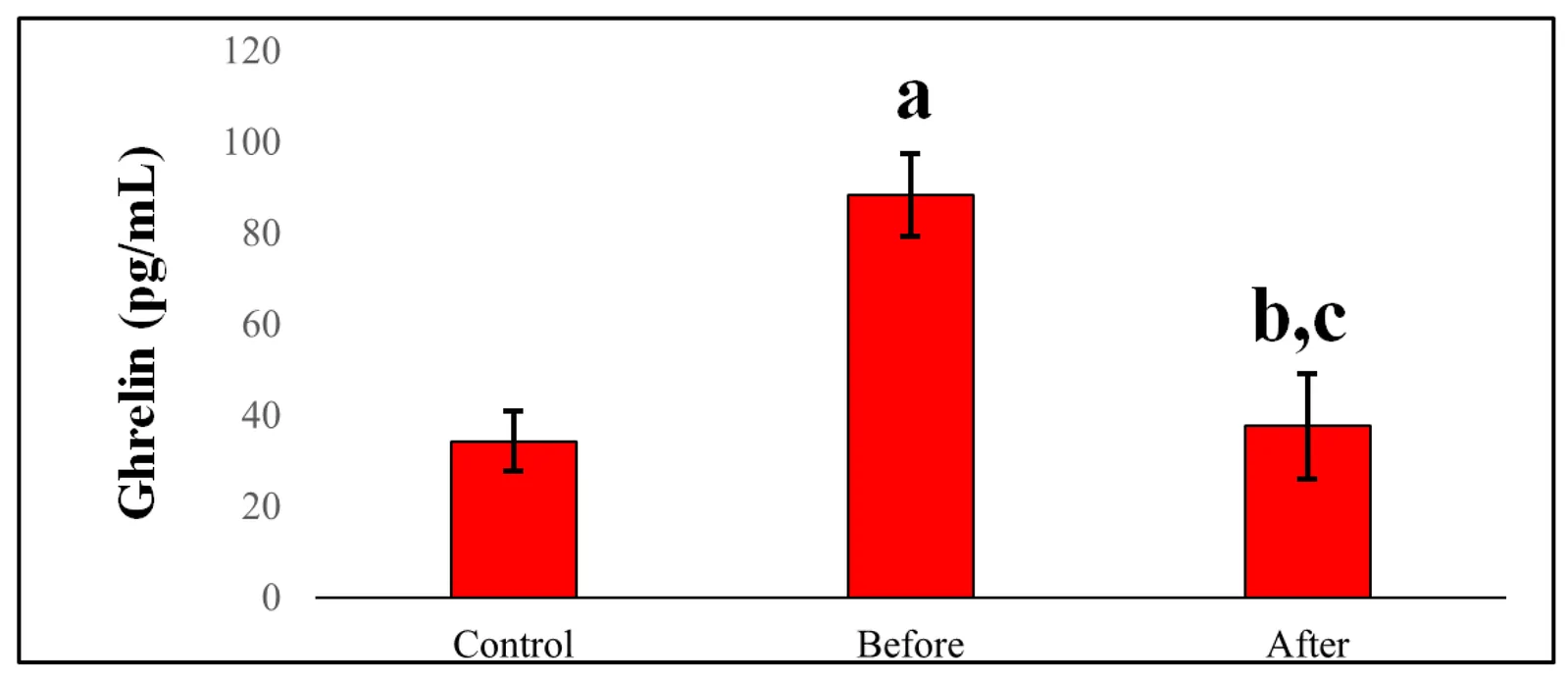
Plasma GHR levels in healthy controls and patients with superficial venous thrombosis before and after LMWH. Data are presented as mean ± SD, and error bars represent standard deviations. GHR: Ghrelin. LMWH: low-molecular-weight heparin. a: Healthy control group versus pre-treatment group (*p* < 0.001); b: Healthy control group versus post-treatment group (*p* = 0.430); c: Pre-treatment group versus post-treatment group (*p* < 0.001).

**Figure 5:**
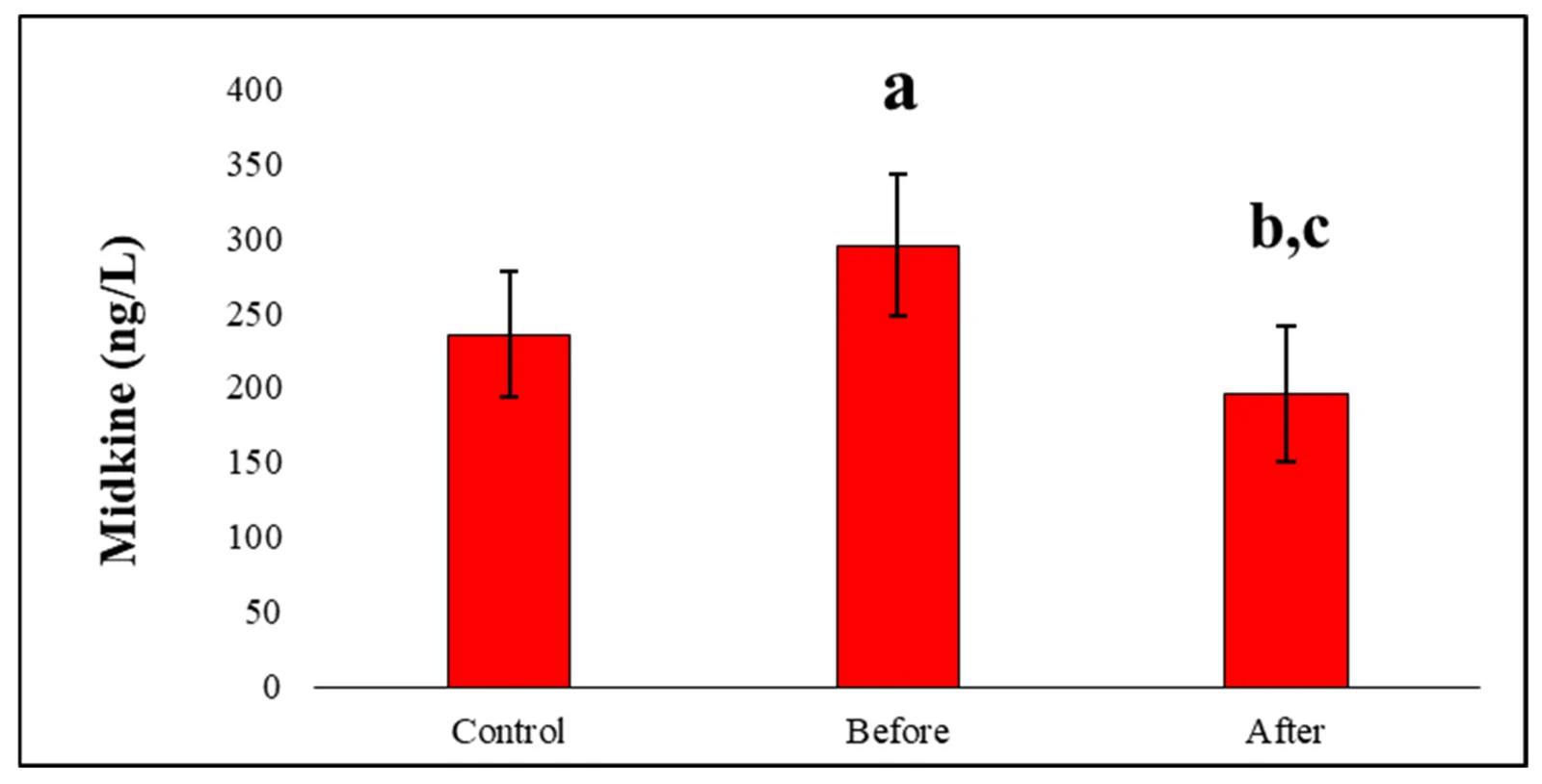
Plasma MK levels in healthy controls and patients with superficial venous thrombosis before and after LMWH. Data are presented as mean ± SD, and error bars represent standard deviations. MK: midkine. LMWH: low-molecular-weight heparin. a: Healthy control group versus pre-treatment group (*p* = 0.002); b: Healthy control group versus post-treatment group (*p* = 0.002); c: Pre-treatment group versus post-treatment group (*p* < 0.001).

**Figure 6:**
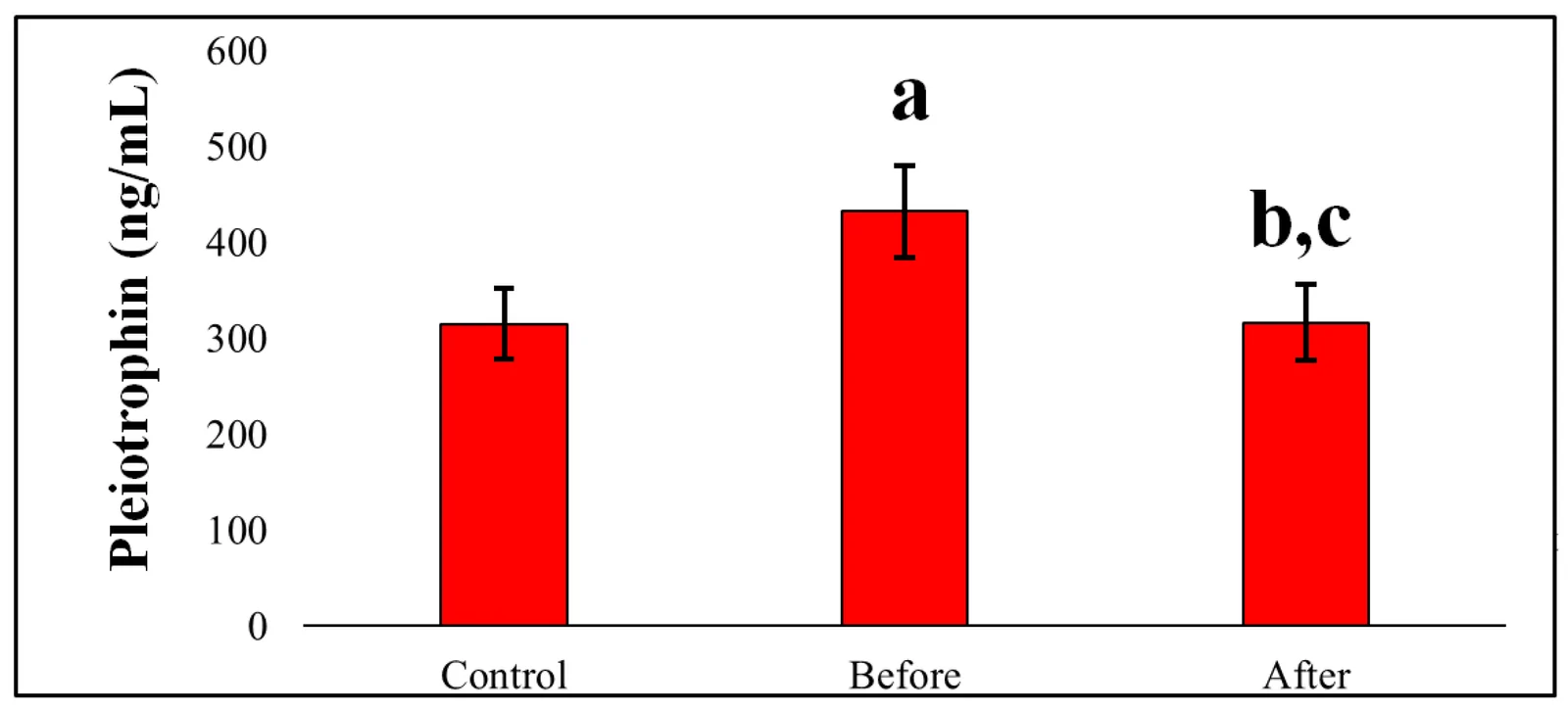
Plasma PTN Levels in Healthy Controls and Patients with superficial venous thrombosis before and after LMWH. Data are presented as mean ± SD, and error bars represent standard deviations. PTN: pleiotrophin. LMWH: low-molecular-weight heparin. a: Healthy control group versus pre-treatment group (*p* = 0.002); b: Healthy control group versus post-treatment group (*p* = 0.614); c: Pre-treatment group versus post-treatment group (*p* < 0.001).

**Figure 7:**
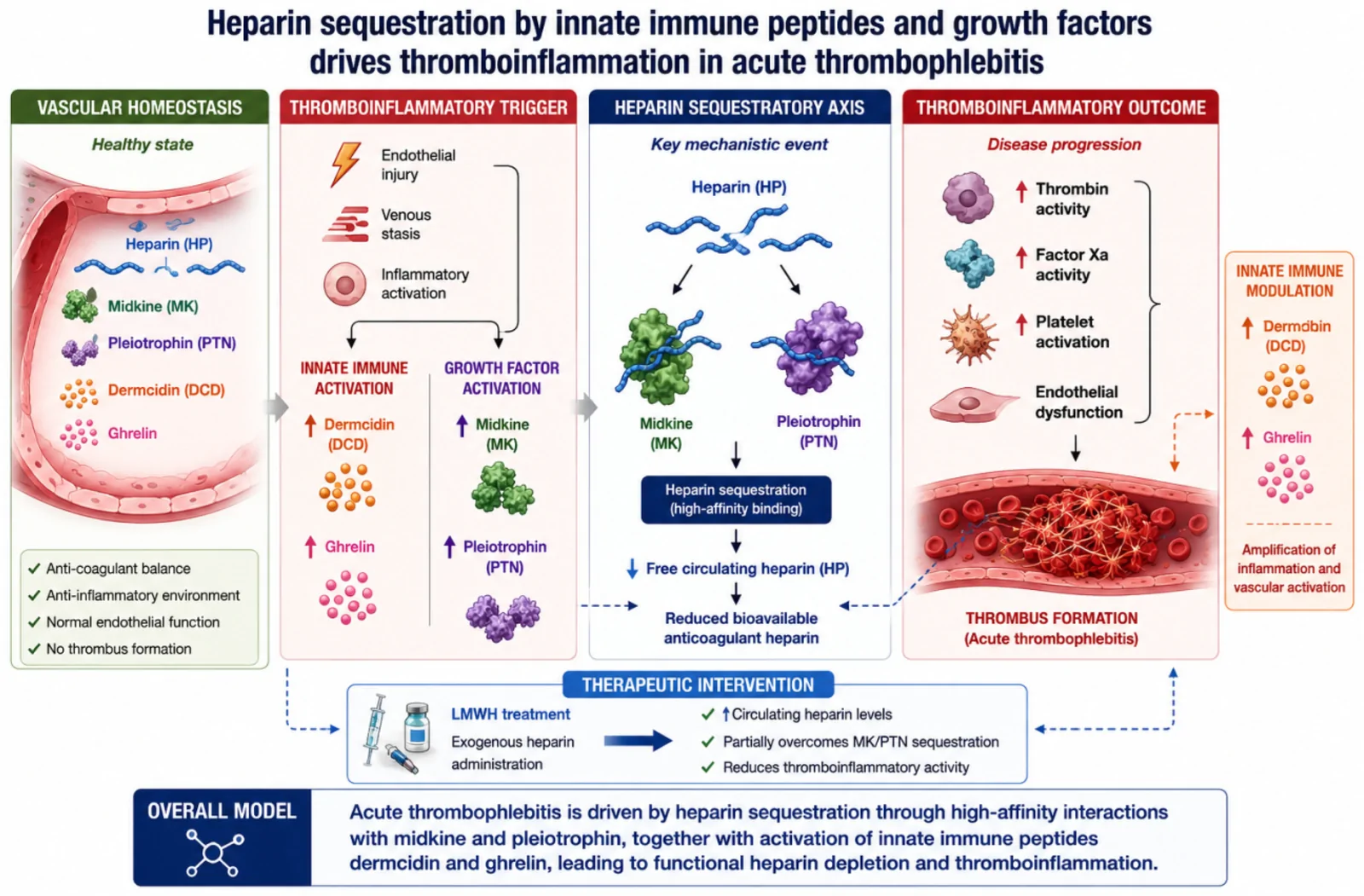
Proposed Mechanistic Model of the Ghrelin–Dermcidin–Heparin–Midkine–Pleiotrophin Axis in Thrombophlebitis (proposed mechanistic model was generated with assistance from ChatGPT (OpenAI) and finalized by the first author).

**Table 1:** Baseline Demographic and Anthropometric Characteristics of Patients with superficial venous thrombosis (SVT) and Healthy Controls.

Parameters	Control Group (*n* = 28)	SVT Group (*n* = 30)
Age (years)	56 ± 11.4	52 ± 8.7
BMI (kg/m^2^)	25.8 ± 2.1	26.7 ± 3.2
Smoking status (female/male)	4/6	6/8

BMI: boBMI: body mass index.

**Table 2:** Etiological Characteristics of Patients with superficial venous thrombosis (*n* = 30).

Etiological Factor	
Postpartum state *n* (%)	12 (40.0)
Idiopathic (no identifiable cause) *n* (%)	11 (36.7)
Cardio-renal-metabolic comorbidity (HD, RD, DM) *n* (%)	5 (16.7)
Trauma-related thrombophlebitis *n* (%)	2 (6.6)

HD: heart disease; RD: renal disease; DM: diabetes mellitus.

**Table 3:** Distribution of Clinical Presentations and Affected Extremities in Patients with superficial venous thrombosis (*n* = 30).

Clinical Symptoms		Affected Extremity	
Pain and swelling, *n* (%)	16 (53.3)	Left extremity *n* (%)	19 (63.3)
Pain and erythema, *n* (%)	6 (20.0)	Right extremity *n* (%)	7 (23.3)
Ecchymosis Swelling and erythema, *n* (%)	8 (26.7)	Bilateral involvement *n* (%)	4 (13.3)
Ecchymosis (bruising), *n* (%)	3 (10.0)	---	---

**Table 4:** Correlations among circulating DCD, GHR, HP, MK, and PTN concentrations in patients with superficial venous thrombosis.

Groups	*p* Value	*r* Value
HP-DCD	0.001	−0.599
HP–GHR	0.001	−0.563
HP–MK	0.114	−0.170
HP–PTN	0.124	−0.165
DCD–GHR	0.001	+0.399
DCD–MK	0.248	+0.124
DCD–PTN	0.042	+0.307
**GHR–MK**	**0.001**	**+0.418**
**GHR–PTN**	**0.002**	**+0.321**
**MK–PTN**	**0.017**	**+0.253**

DCD: dermcidin. GHR: ghrelin. HP: heparin. MK: midkine. PTN: pleiotrophin.

## Data Availability

The datasets generated and/or analyzed during the current study are available from the corresponding author on reasonable request.

## Abbreviations

The following abbreviations are used in this manuscript:

BMI	Body Mass Index
CV	intra-assay Coefficient of Variation
DCD	Dermcidin
DVT	Deep Vein Thrombosis
ELISA	Enzyme-Linked Immunosorbent Assay
GHR	Ghrelin
HP	Heparin
LMWH	Low-Molecular-Weight Heparin
MK	Midkine
NETs	Neutrophil Extracellular Traps
OD	Optical Density
PTN	Pleiotrophin
